# Exploring innovative G-CSF schedules in AML cytarabine-based consolidation through a digital twin study of white blood cell recovery

**DOI:** 10.1371/journal.pone.0329242

**Published:** 2026-01-27

**Authors:** Adrian-Manuel Reimann, Enrico Schalk, Dimitrios Mougiakakos, Thomas Fischer, Sebastian Sager

**Affiliations:** 1 Department of Mathematics, Otto von Guericke University, Magdeburg, Germany; 2 Department of Hematology, Oncology and Cell Therapy, Medical Faculty, Otto von Guericke University, Magdeburg, Germany; 3 Healthcampus Immunology, Inflammation and Infectiology (GC-I3), Otto von Guericke University Magdeburg, Magdeburg, Germany; 4 Center for Health and Medical Prevention – CHaMP, Otto von Guericke University Magdeburg, Magdeburg, Germany; 5 Institute of Molecular and Clinical Immunology, Medical Faculty, Otto von Guericke University Magdeburg, Magdeburg, Germany; 6 Max Planck Institute for Dynamics of Complex Technical Systems, Magdeburg, Germany; Azienda Ospedaliera Universitaria SS Antonio e Biagio e Cesare Arrigo, Alessandria, University of Eastern Pedemont, ITALY

## Abstract

Patients with acute myeloid leukemia (AML) are at high risk for life-threatening infectious complications due to chemotherapy-induced leukopenia. In contrast to other malignancies, prophylactic administration of granulocyte colony-stimulating factors (G-CSF) is not routinely done in AML. However, recently, clinical trials showed that administration of G-CSF post consolidation chemotherapy leads to faster white blood cell (WBC) recovery and to fewer infections. We investigated novel G-CSF schedules using a mathematical model trained with retrospective clinical data (digital twins) consisting of high-frequency measurements of 65 patients. We evaluated 323 different treatments varying in start and duration of G-CSF administration by examining the impact on the occurrence and duration of leukopenia. For each treatment, we used the same 65 digital twins, plus an additional 100 artificial patients for uncertainty quantification, receiving 3 consolidation cycles each. We lumped the 323 treatments into 4 clusters: NO-G-CSF, PRE-G-CSF, SIM-G-CSF, and POST-G-CSF for schedules without G-CSF, that terminated G-CSF before consolidation chemotherapy started, that overlapped on at least one day, and that started post chemotherapy, respectively. The numerical simulations resulted in substantial differences in the occurrence of leukopenia: 88% of all consolidation cycles with leukopenia for NO-G-CSF, 98% for SIM-G-CSF, 45% for POST-G-CSF, and 28% for PRE-G-CSF. Administration of G-CSF before chemotherapy may thus significantly enhance the efficacy of AML consolidation therapy and, therefore, warrants clinical investigation.

## Introduction

The primary goal of therapy in patients with acute myeloid leukemia (AML) is remission induction. In general, after achieving remission, patients with a relatively low recurrence risk of <35–40% [1] receive consolidation with intermediate-dose (ID) or high-dose (HD) Ara-C [2], which, as a drug, disrupts DNA synthesis, particularly in rapidly dividing cells, whereas high-risk patients undergo hematopoietic stem cell transplantation. Depending on the molecular/cytogenetic profile, the addition of other agents in induction and consolidation, such as midostaurin for FLT3-mutated patients [3,4], was shown to improve prognosis. Patients with AML are at increased risk for developing life-threatening infectious complications, such as pneumonia or bloodstream infections, both as a result of AML-induced neutropenia and intensive treatment with cytotoxic (chemo-) therapy [5]. Patients with AML have a very high risk (>80%) of infections, which are the primary cause of death during therapy in >70% of cases and are one of the most common reasons for transfer to the intensive care unit [6,7].

Neutropenia, defined as an absolute neutrophil count of <500/µL, or in general, leukopenia characterized by a white blood cell [WBC] count <1000/µL as a surrogate [8], is the main risk factor for infectious complications in patients with AML [9]. The incidence and severity of infections depend on the depth and duration of neutropenia [5,10]. In patients with AML, microbiologically documented infections tend to occur at the time point of the neutrophil nadir, while clinically documented infections generally occur some days earlier [11]. Another important risk factor of infection is the degree and timing of gut mucosal damage. Neutropenia aggravates this complication and may result in the clinical presentation of neutropenic enterocolitis [12].

For some non-myeloid malignancies with high-dose chemotherapy treatments, administration of granulocyte colony-stimulating factors (G-CSF) is recommended; however, the most effective timing is still under discussion [13]. In comparison, prophylactic administration of G-CSF is not generally recommended in AML [14], although it is capable of reducing both the duration and the severity of neutropenia [15]. It remains controversial due to a theoretically increased risk of relapse and indeterminate impact on neutropenic complications [16]. Results from clinical trials showed that in AML consolidation therapy, administration of Ara-C on days 1, 2, and 3 (Ara-C-123) was superior in comparison to Ara-C on days 1, 3, and 5 (Ara-C-135) because of faster hematologic recovery and a lower incidence of infections. Interestingly, pegfilgrastim, a depot G-CSF formulation, when administered a few days after the consolidating chemotherapy, further reduced the infection rate [17,18]. However, improved survival was not seen [17,18].

In addition, previously, G-CSF has been applied concurrently with AML induction chemotherapy as a priming approach to drive leukemic stem cells into S-phase of the cell cycle. This approach aimed to increase the sensitivity to S-phase specific chemotherapy such as cytarabine. However, the results of several randomized trials did not justify further application of this strategy [19]. For example, neutrophils regenerated with a delay (P = .007) in patients assigned to a concurrent G-CSF treatment regimen [20]. Also in our simulations, the concurrent, overlapping administration of cytarabine and G-CSF during consolidation therapy did result in delayed WBC recovery times. We considered novel G-CSF schedules that, like priming, are applied before consolidation chemotherapy, but do not overlap with induction chemotherapy. We hypothesized that in AML consolidation therapy, such innovative G-CSF schedules have the potential to substantially reduce the nadir and the duration of leukopenia and may significantly improve the outcome of AML treatment. Therefore, we investigated novel G-CSF schedule variants and examined the induced changes in the occurrence and duration of leukopenia upon AML consolidation therapy. Motivated by the large number of existing G-CSF schedules, we used digital twins, specified in the upcoming section and the supplementary material S1 File, to fully explore the wide spectrum of different combinations regarding both the starting point of administration and the duration of G-CSF application.

## Materials and methods

Referring to the different possible concepts for digital twins in oncology [21], we define a digital twin as a mechanistic mathematical model with model parameters that allow personalization with clinical data. In this study, we used 65 digital twins that are specified via six individual model parameters each. Both the model and the parameters are described in the supplementary material S1 File. The training data used to establish these digital twins were obtained from a cohort of patients who were heterogeneous with respect to Ara-C administration and timing, whether or not and how lenograstim as a G-CSF was administered, age (median 58.5 years), and number of observed longitudinal WBC counts, with a total of 1869 observations. Variance of G-CSF schedules was not a focus of AMLSG 12−09, and none of the patients received a non-standard PRE-G-CSF or SIM-G-CSF treatment.

In addition to the mathematical model with personalized model parameters specifying a digital twin, four more clarifications are necessary. First, under “Model Validation” we describe the strategies implemented to ensure the model’s reliability and to support the plausibility of its predictions. Second, in “Treatment choices” we discuss the degrees of freedom associated with the clinical treatment, i.e., the administration of Ara-C and G-CSF. Third, in “Considered scenario” we specify the length of the simulation horizon and our choice of the initial values of all differential states. Fourth, in “Simulating and predicting treatment outcomes” we explain how we evaluated WBC recovery based on the numerical solution of differential equations (simulation). In summary, our approach allows us to specify G-CSF schedules (at what times, how much is administered) and obtain corresponding WBC recovery times and blast ratios for all simulated CCs of all digital twins.

### Model validation

Digital twins can predict, within a margin of uncertainty, the outcomes of different treatment strategies. However, forecasting outcomes for interventions not represented in the training data—such as non-standard G-CSF schedules—remains inherently challenging. The dataset we used in this study contains approximately 30 observed time points per patient, making it considerably more densely sampled than most AML data sources and therefore particularly suitable for training digital twins. The mechanistic model integrates both pharmacodynamic and pharmacokinetic components, as detailed in Supplementary Material S1 File. This modeling framework preserves biological interpretability, a key prerequisite for translating predicted treatment effects into a clinically meaningful context. Extensive validation work has been conducted, suggesting that the model is robust both structurally—reflecting mechanistic consistency across treatment regimes—and quantitatively, as evidenced by close agreement between simulated and observed WBC and blast trajectories. Specifically, a previous study [22] demonstrated that the distributions of WBC recovery times in this 65 digital twin cohort did not differ significantly from those of larger clinical cohorts, even for treatments only partly included in the training set. Moreover, [23] showed that digital twins derived from this model and data were capable of reproducing individual treatment outcomes.

To further strengthen the validity of our approach, we conducted an uncertainty quantification by generating 100 artificial patients from the digital twins through propagation of random variations in model parameters. The analysis, which includes a comparison of relevant performance indicators to assess the model robustness, is described in detail in Supplementary Material S1 File. Owing to its scope and depth, the analysis is presented entirely in the supplement, as it extends beyond the primary focus of this manuscript. The underlying system of differential equations, together with detailed information on the retrospective, longitudinal dataset—including WBC count measurements, treatment data, and leukemic blast dynamics—is also provided in Supplementary Material S1 File.

### Treatment and schedule choices

In the interest of a clear focus on different G-CSF schedules, chemotherapy treatment was specified to the administration of high dose 3 g/m^2^ (n = 30) or intermediate dose 1-1.5 g/m^2^ (n = 35) Ara-C on days 1, 2, and 3 of each consolidation cycle (CC), with the dosage chosen according to the underlying clinical data to reduce extrapolation errors of the mathematical model. Throughout, as it did not affect the qualitative result, a BSA of 2 m^2^ was assumed. Ara-C was administered twice a day at 8 am and 8 pm consistently across all simulations. The length of all CCs was fixed to 35 days. The length of CCs has an important impact on WBC recovery time [22]. Yet, for each choice we considered other than 35 days, the results concerning relative improvements due to G-CSF administration were qualitatively similar, as was also the case for Ara-C135. We fixed the dosage of G-CSF to one infusion of 263 µg per day at 12 pm and focused on different timings and lengths of G-CSF administration. We defined the starting point “s” of G-CSF treatment relative to the first CC day on which the first dosage of Ara-C is administered: G-CSF starting day 0 indicates a simultaneous start of G-CSF and Ara-C treatment on the first day of each CC. We varied the starting day of G-CSF treatment from −23–21 in steps of 2 days and the duration “l” of treatment from 1 to 14 in steps of 1 day. Adding no G-CSF treatment (i.e., l = 0), this resulted in n = 23 × 14 + 1 = 323 treatments.

To be able to assess whether administration of G-CSF before, during, or after the administration of chemotherapy is beneficial in comparison to not giving G-CSF, we clustered the 323 treatments into 4 classes. Using s as the start day and l as the duration length, we defined POST-G-CSF as the union of all treatments with s>=3 (140 schedules), PRE-G-CSF as the union of all treatments with l + s < 1 (119 schedules), and SIM-G-GCSF as the union of all remaining treatments (63 schedules). NO-G-CSF contains only 1 schedule, the one with l = 0.

### Considered scenario

**Fig 1:**
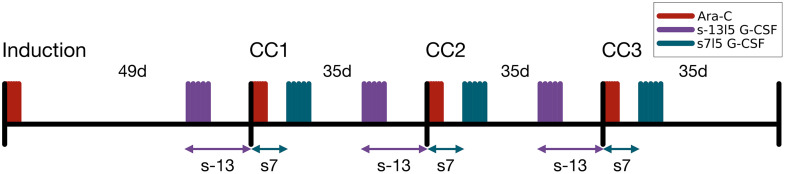
Illustration of the simulation specifics. The first interval of 49 days allows the consideration of G-CSF treatments starting before Ara-C treatment, i.e., before a consolidation cycle (CC) begins. This is necessary for some of the treatments with the start of G-CSF s < 0. Considering the time interval of 154 days, Ara-C is always administered on days 1, 2, 3 (induction), 50, 51, 52 (CC1), 85, 86, 87 (CC2), 120, 121, and 122 (CC3). The G-CSF administration depends on the specifications of s and **l.** For example, for s = −13 and l = 5, it is administered on days 37-41 (CC1), 72-76 (CC2), and 107-111 (CC3).

**Fig 2:**
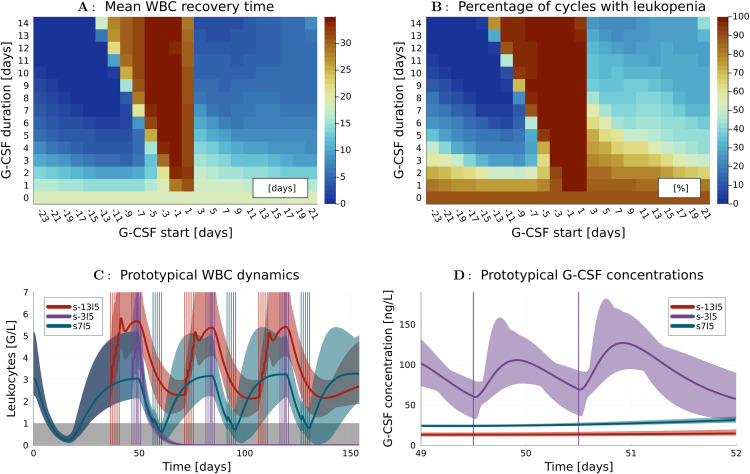
Simulated WBC recovery after chemotherapy and G-CSF treatment. **A** The heatmap illustrates the WBC recovery time in days for different G-CSF schedules. They differ in the start s of the treatment (x-axis) relative to the beginning of Ara-C administration and in the number of consecutive days G-CSF is administered (G-CSF duration l on the y-axis). E.g., a G-CSF start s = −3 denotes G-CSF administration starting three days before Ara-C administration, and a duration of l = 5 denotes that G-CSF is administered for 5 consecutive days. The color of each cell encodes mean values taken over 195 simulated CC of 65 digital twins for a particular G-CSF treatment. The mean is lowest (blue) for treatments on the left hand side of the plot, corresponding to early administration of G-CSF before chemotherapy. The mean is slightly higher (light blue) on the right hand side of the plot, corresponding to G-CSF treatments after termination of chemotherapy. The G-CSF treatments overlapping with chemotherapy show the worst performance of more than 30 days (red). In addition, a general trend that longer durations lead to faster hematologic recovery can be observed. **B** A similar heat plot shows a color encoding of the percentages of the 195 CCs for each G-CSF treatment, in which a leukopenia occurred. The heat plot has a similar geometry to the one shown in Fig 2A, with a more emphasized difference between the percentages close to 0% on the left hand side and the percentages ranging between 30% and 90% on the right hand side. **C** For a prototypical digital twin and three exemplary G-CSF treatments of the same duration l = 5, the G-CSF timing (vertical bars) and the corresponding time trajectories of WBC counts, together with an uncertainty range as specified in the supplementary material S1 File, are plotted. The red trajectory corresponding to the early G-CSF treatment s-13l5 oscillates between values well above the critical leukopenia region in grey. The violet trajectory corresponding to a G-CSF treatment overlapping with chemotherapy s-3l5 temporarily increases the WBC count, but shows a drastic reduction of WBC, from which it does not recover in the simulated time horizon. The blue trajectory corresponding to G-CSF after Ara-C, s7l5, shows temporary increases of WBC counts, but also time periods of leukopenia. **D** Corresponding G-CSF concentrations are plotted for days 49-52 when chemotherapy is administered. The increased G-CSF values for s-3l5 result in a stronger impact of Ara-C on the decrease of WBC observed in Fig 2C.

We considered a time horizon of 154 days, consisting of a first interval of 49 days, followed by three CC of 35 days each. The first period of 49 days was used to simulate the effect of a previous chemotherapy treatment (such as induction therapy) on the differential states. This scenario was meant to reflect the clinical setting in which consolidation therapy follows upon induction therapy, and the hematologic system can be expected to be in a phase of recovery. The concept is visualized in Fig 1. In Fig 2C, a prototypical WBC trajectory is shown over the time period from day 1–154 for G-CSF treatments with three different administration timings, which are also plotted.

### Simulating and predicting treatment outcomes

We calculated treatment outcomes for all digital twins by simulation using the *Rosenbrock W6S4OS* method from *DifferentialEquations.jl*. We used the WBC recovery time for every CC as a key performance indicator for leukopenia severity. WBC recovery time is a clinically established parameter.

In summary, we obtained results for 323 different G-CSF treatments and 65 digital twins, and 3 consecutive CCs, each. For all, we extracted the WBC recovery times. The WBC recovery time was set to 0 if the WBC was above 1000/*µL* at all times, i.e., if no leukopenia occurred.

### Ethics statement

The training data used in this study were obtained retrospectively from the phase II AMLSG 12−09 randomized-controlled trial (RCT; *n* = 44), collected between 2010-10-11 and 2012 [24], and from clinical chart records obtained between 2008 and 2015 from the Magdeburg University Hospital, Germany (*n* = 21). The phase II AMLSG 12−09 protocol was approved by the Ethics Committee and is registered at clinicaltrialsregister.eu (EudraCT Number: 2009-016142-44) and clinicaltrials.gov (ClinicalTrials.gov Identifier: NCT01180322). Written informed consent was obtained from all patients treated within this trial. The study was completed on 02.10.2016 and published on 22.10.2017 [24]. We had no access to information that could identify individual participants from this trial. The second study used was approved by the ethics committee of the Magdeburg University Hospital (approval no. 124/15). Data from this trial were pseudonymized. The data was accessed for research on 18.06.2018. Given the nature of a retrospective chart review study and using routine clinical data, written informed consent was not required within the study. No individual patient can be re-identified from any of the information presented in this manuscript or its supplementary materials.

## Results

To illustrate the distribution of WBC recovery times, Fig 2A shows the mean (concerning the 195 CCs of 65 digital twins) WBC recovery times resulting in one value for each of the 323 different G-CSF treatments. Each cell in the heat plot corresponds to a particular G-CSF treatment, specified via starting day s on the horizontal axis and duration length l on the vertical axis, and corresponds to the mean WBC recovery time indicated by the heatmap.

One can visually discern three distinct classes among the 322 G-CSF treatments, aligning with the pre-defined sets PRE-G-CSF, SIM-G-CSF, and POST-G-CSF. SIM-G-CSF, the triangular-shaped area with many red cells, shows the poorest performance concerning WBC recovery time, even worse than NO-G-CSF (the cell with l = 0). The POST-G-CSF treatments with starting day s>=3 correspond to the light bluish area on the right-hand side of the red triangle. A good choice here is s = l = 7, resulting in a mean WBC recovery time of 6 days. The PRE-G-CSF treatments correspond to the dark bluish area on the left-hand side of the red triangle. An exemplary good choice for PRE-G-CSF is s = −13, l = 7, resulting in a mean WBC recovery time of 0 days.

Fig 2B shows, in a similar way, the percentages of leukopenic CCs, i.e., for which the WBC was at least at one time point below 1000/*µL*. The visual separation in the heat plot is identical to Fig 2A. Again, SIM-G-CSF treatments form a triangle, affecting almost all digital twins with up to 100% leukopenia occurrences. The situation is drastically better for POST-G-CSF treatments, which reduce the incidence of leukopenia to percentages between 30% and 90%, approximately. A clear trend indicates that the longer G-CSF is administered, the lower the occurrence of leukopenias, with decreasing percentages observed towards the upper end of the subplot. This lowering risk of leukopenias is mostly due to the strong increase of WBC that carries over to the next simulated CC, resulting in the Chemotherapy not lowering the WBC below 1000/*µL* after the first CC. The PRE-G-CSF treatments have the potential to reduce the percentage of leukopenias to 0%, e.g., for s = −13 and l = 7 and longer durations, since the overlapping effect observed in the top right area for POST-G-CSF is already present for the first CC when considering PRE-G-CSF.

To quantify these visual impressions, we also looked at mean values for all treatments in the four classes. The numerical simulations resulted in different outcomes concerning mean WBC recovery times in days and percentage of CCs with leukopenia: 19 days and 88% for NO-G-CSF, 32 days and 98% for SIM-G-CSF, 8 days and 45% for POST-G-CSF, and 6 days and 28% for PRE-G-CSF.

To get a better understanding of the WBC recovery in response to the different treatment schedules, Fig 2C shows for one prototypical digital twin and an uncertain (see supplementary material S1 File for details) evolution of WBC counts over time for three different G-CSF application schedules. The length of all treatments is identical with l = 5 and the starting days s = −13, s = −3, s = 7 are shifted by 10 days, such that we have one example from the PRE-G-CSF, SIM-G-CSF, and POST-G-CSF group, respectively. The early administration of G-CSF at s = −13 leads to oscillations in the range between 3000/*µL* and 6000/*µL*. For starting date s = −3 with an overlap between G-CSF and Ara-C administration, the WBC count drops dramatically. For the schedule to administer G-CSF after Ara-C, here at s = 7, we observe a temporary increase in the WBC count, which does not result in completely avoiding leukopenia, though.

Fig 2D zooms into the critical time horizon between days 49 and 52 when Ara-C is administered and visualizes the concentration of G-CSF in the body. One observes that, due to the administration of G-CSF, the concentration for s = −3 is significantly higher than for the s = −13 and s = 7 treatments. Thus, the increased G-CSF exposure at a time point when Ara-C effects peak leads to increased hematopoietic toxicity and the delayed but steep decline of WBC shown in Fig 2C.

## Discussion

### Main results

Our main hypothesis was that novel schedules of G-CSF application employing early administration prior to consolidation chemotherapy might result in fewer and less severe leukopenias in AML. Leukopenia in AML is generally viewed as a measure of neutropenia [8]. As apparent from Figs 2A and 2B and from the mean values of 28% CCs with leukopenias for PRE-G-CSF in comparison to 45% for POST-G-CSF, 98% for SIM-G-CSF, and 88% for no G-CSF at all, this hypothesis seems plausible.

The advantage of early G-CSF administration is not a simple consequence of more time for WBC to grow, but of more complex dynamics. This can be seen by looking at the oscillatory nature of the system, which would result in WBC going back to a steady state value in the long run (compare Fig 2C), and by the non-monotonicity observable for the SIM-G-CSF schedules.

A secondary result is that for both PRE-G-CSF and POST-G-CSF treatments, longer durations provide better results with respect to WBC recovery. Disadvantages of prolonged G-CSF administration, such as increased risk of adverse effects or economic costs, are not considered here.

### The study’s rationale

Although classical consolidation therapy has been based on Ara-C for decades, there are open questions concerning the optimal number and duration of cycles as well as dosage and timing of Ara-C administration [17,18,25]. If and how G-CSF should be prophylactically administered remains an open question and is not generally recommended in patients with AML [14]. An examination of US American guidelines [26] references the FDA label stating that pegfilgrastim should be avoided 14 days before or within 24 hours of administration of myelosuppressive chemotherapy [27] and attributes this to the theoretical potential to increase the toxicity of chemotherapy to myeloid progenitor cells following growth factor stimulation [28]. Studies reported a strong increase in the incidence of severe neutropenia. In general, caution is warranted when comparing different G-CSF formulations, such as lenograstim and pegfilgrastim, as potential differences in pharmacokinetics, dosing schedules, and clinical effects cannot be excluded. However, our findings suggest that certain similarities in their behavior may be inferred. For example, we observed an increase in the number and severity of leukopenias, also in our simulations. These outcomes are well matched by the red triangles in Fig 2A and 2B, corresponding to SIM-G-CSF, and are plausible by looking at the physiological properties encoded in the mathematical model. Large G-CSF concentrations stimulate cell proliferation, which leads to a strong increase in chemotherapy toxicity when administered at the same time. The recommended 24h margin aligns well with our results for s = 3. However, our results (left-hand side of the red triangle) show that a safety distance of 2 days is enough when comparing the end of G-CSF administration and the beginning of chemotherapy. This gives ample room for treatment improvements when compared to the recommended 14 days [26].

Regarding the potential for additional prophylactic G-CSF administration applied after chemotherapy, there are contradictory results in the literature. Only minor improvements in the depth and duration of nadir and thus in infection and mortality rates have been reported [29,30]. In contrast, recent clinical studies [17,18] and a study based on digital twins [22] reported a significant shortening of WBC recovery times of approximately 2 days due to G-CSF administration after chemotherapy.

For other malignancies, the timing of G-CSF is also under current debate. E.g., for patients with lung cancer receiving chemotherapy weekly, the question of same-day versus next-day administration of G-CSF has been discussed [31,32]. Note that this discussion corresponds to a discussion of the exact boundary between SIM-G-CSF and POST-G-CSF in our setting, i.e., to the vertical lines around s = 3 in Figs 2A and 2B. The result that both seem to have a similar, beneficial impact on the reduction of febrile neutropenia [31,32] is consistent with our simulation results. Note that logistical aspects of different G-CSF administrations have also been discussed in this context [33]. However, direct comparison to the AML setting should be made with caution, as treatment response, underlying mechanisms, and treatment guidelines generally differ. If increased duration or dosage of G-CSF is considered, corresponding to better performing upper rows in Figs 2A and 2B, possible side effects of overdosage [34] and economic and logistic costs also need to be considered.

### Clinical feasibility

It is important to note that the PRE-G-CSF schedules with s-13 correspond to the POST-G-CSF schedules using s22 of the preceding cycle, assuming a cycle length of 35 days. This relationship illustrates that for s-13 sufficient time for hematological recovery is available within a consolidation cycle before the subsequent PRE-G-CSF treatment is administered ensuring clinical feasibility (see Fig 1). The primary distinction between s-13 and s22 lies in the timing of G-CSF administration across consolidation cycles: in s-13, the first cycle already includes a PRE-G-CSF treatment, which is not present in s22. Conversely, in s22, a POST-G-CSF treatment follows hematological recovery in the final cycle, which is absent in s-13. Such translations between PRE- and POST-/SIM-G-CSF schedules should be carefully considered when evaluating clinical feasibility. As our simulations include PRE-G-CSF treatments up to s-23, it is evident that not all simulated regimens are feasible in clinical practice. Nevertheless, they are presented to illustrate general behavioral trends and treatment response patterns that may inform future investigations (see Fig 2).

### Choice of methods

There are different purposes and ways of using digital twins [21]. Here, we decided to use a mechanistic mathematical model that was trained and cross-validated with clinical data and used (partially) in a series of previous publications [22,23,35–37]. It focuses on aspects that are relevant for the open questions mentioned above, i.e., myelosuppression, pharmacokinetics, and -dynamics of Ara-C and G-CSF (lenograstim). Advantages in comparison to deep learning-based approaches are interpretability, better extrapolation properties, and a reduced amount of necessary training data. The simulated outcomes are, of course, uncertain and have to be verified by clinical studies. As none of the 65 patients included in the training dataset received a non-standard PRE-G-CSF or SIM-G-CSF regimen, the reliability of model predictions for such treatment schedules may be limited. A detailed description of the model validation procedure, including considerations for non-standard treatment scenarios, is provided in the Model Validation subsection of Materials and Methods. High dose chemotherapy is a large perturbation of the bone marrow microenvironment, the hematopoietic cells, and the cytokine feedback. Therefore, it is not clear if the implied assumption that hematopoietic cells before, during, and after chemotherapy respond identically to external G-CSF administration is valid. Further validation of the presented findings in independent patient cohorts, ideally including non-standard G-CSF schedules, would be an important next step to substantiate and generalize the model predictions. Therefore, all results need to be interpreted very carefully. Yet, our goal was not to predict individual responses of individual patients as well as possible, but rather to obtain a representative cohort of patients, in which such uncertainties might also balance out. The smoothness of the results with respect to G-CSF starting date and duration in Fig 2, also for the supporting uncertainty quantification results in the supplement S1 File, makes it, in our opinion, a clear indication that it is worthwhile to follow these indications with a validating clinical trial.

### Clinical outlook

We propose to explore the investigated early administration of G-CSF in a clinical trial or a mouse clinical trial. We anticipate that our results based on digital twins will translate qualitatively into clinical reality. Initiating G-CSF administration early in the treatment course may enhance the therapeutic effectiveness of AML consolidation therapy.

However, improvements of classical consolidation therapy have to be seen in the context of the trend to an increase in allogeneic hematopoietic stem cell transplantation after induction therapy and to use targeted therapies involving, e.g., FLT3- or IDH-inhibitors. A development of digital twins able to represent these treatments might help to design even better leukemia therapies. For the future, we believe that investigation of novel G-CSF schedules in digital twins may offer major improvements in WBC recovery and thus in hematologic toxicity in a wide variety of chemotherapy schedules of other hematologic and solid malignancies.

## Supporting information

S1 File Extended model formulations, parameter definitions, and technical derivations complementing the main text. (PDF)
